# Amyloid-related imaging abnormalities in Japanese patients with Alzheimer’s disease treated with Lecanemab: A real-world study

**DOI:** 10.1016/j.tjpad.2026.100562

**Published:** 2026-04-04

**Authors:** Ryosuke Shimasaki, Masanori Kurihara, Taro Bannai, Keiko Hatano, Fumio Suzuki, Aya Midori Tokumaru, Kenji Ishii, Ryoko Ihara, Atsushi Iwata

**Affiliations:** a Department of Neurology, Tokyo Metropolitan Institute for Geriatrics and Gerontology, Tokyo, Japan; b Integrated Research Initiative for Living Well with Dementia, Tokyo Metropolitan Institute for Geriatrics and Gerontology, Tokyo, Japan; c Department of Diagnostic Radiology, Tokyo Metropolitan Institute for Geriatrics and Gerontology, Tokyo, Japan; d Research Team for Neuroimaging, Tokyo Metropolitan Institute for Geriatrics and Gerontology, Tokyo, Japan

**Keywords:** Alzheimer’s disease, Lecanemab, Aria, Japanese cohort, Real-world data

## Abstract

**Background:**

Although clinical trials have suggested a lower incidence of adverse events associated with Lecanemab in Asian populations compared to global cohorts, longitudinal real-world data across broader clinical indications are necessary to confirm these findings in routine practice.

**Objectives:**

This study aimed to provide real-world evidence regarding the safety profile of Lecanemab in Japanese patients in a clinical setting.

**Design:**

A real-world observational study with a follow-up period of up to 18 months.

**Setting:**

A single center in Japan.

**Participants:**

We included 120 Japanese patients who received Lecanemab between December 2023 and November 2025 and underwent at least one brain MRI before the fifth infusion.

**Measurements:**

Safety outcomes included amyloid-related imaging abnormalities (ARIA), infusion-related reactions (IRRs), and treatment discontinuation.

**Results:**

The mean age was 74.2 ± 7.9 years, and 89 (74%) were female. The majority of patients (88%) had a baseline CDR global score of 0.5. During follow-up, 81 patients completed the 12-month assessment. ARIA occurred in 24 patients (20%); ARIA-E with or without ARIA-H occurred in 5 patients (4%), and isolated ARIA-H occurred in 19 patients (16%). Crucially, no patients experienced symptomatic ARIA. All patients with ARIA-E who had available *APOE* data were ε4 carriers. Patients with ARIA had significantly lower baseline MMSE scores (*p* = 0.04), alongside non-significant trends toward higher plasma GFAP levels (*p* = 0.11) and higher deep white matter Fazekas scores (*p* = 0.05). IRRs occurred in 34 patients (28%), all of which were mild. Treatment was discontinued in 19 patients (16%), mainly due to disease progression (*n* = 8).

**Conclusion:**

In this Japanese AD cohort, Lecanemab demonstrated a manageable safety profile in a real-world setting. In exploratory analyses, potential trends toward a higher frequency of ARIA were observed in patients with lower MMSE scores, higher plasma GFAP levels, and higher Fazekas scores, underscoring the importance of individualized risk assessment prior to therapy.

## 1 Introduction

Alzheimer’s disease (AD) is a progressive neurodegenerative disorder pathologically characterized by the accumulation of amyloid β (Aβ) and tau protein aggregates forming neurofibrillary tangles (NFT) in the brain [1].

Lecanemab, an anti-amyloid monoclonal antibody targeting soluble Aβ protofibrils [2,3], has consistently demonstrated its ability to slow cognitive and functional decline in early AD [4,5]. In Japan, Lecanemab received regulatory approval on September 25, 2023, and was commercially launched on December 20, 2023. Following its launch, Lecanemab therapy was initially restricted to a limited number of specialized institutions. Since then, the expansion of institutional accessibility has led to a steady increase in the number of patients treated. As the clinical use of Lecanemab has expanded, it has become increasingly important to monitor and appropriately manage adverse events associated with anti-amyloid monoclonal antibodies. Among 1612 individuals treated with Lecanemab in the core or open-label extension of Clarity-AD, four deaths were considered possibly related to the study medication [4,6]. Although treatment is conducted in accordance with the national optimal use guidelines in Japan (OUG) [7,8], the need for careful evaluation of safety is even greater in real-world clinical practice, where protocols may be less strictly adhered to than in controlled clinical trials.

Adverse events associated with anti-amyloid monoclonal antibodies have been reported, consisting primarily of amyloid-related imaging abnormalities (ARIA) and infusion-related reactions (IRRs) [2,9]. ARIA includes cerebral edema or effusion (ARIA-E) and cerebral hemorrhage or hemosiderin deposition (ARIA-H). The number of apolipoprotein E (*APOE*) ε4 alleles is well established as an important determinant of the risk of developing ARIA [10]. This association has also been observed in real-world data [11]. Furthermore, a previous study has demonstrated that the incidence of ARIA and IRRs is lower in Asian participants than in the overall study population [5], suggesting potential differences in patient risk profiles. However, real-world data specifically characterizing the safety profile in Japanese clinical populations remain limited.

This study aimed to evaluate the safety profile of Lecanemab in Japanese patients at a single center, providing real-world evidence from routine clinical practice and characterizing individual patient risk.

## 2 Methods

### 2.1 Participants

A total of 120 patients meeting the eligibility criteria for Lecanemab therapy were enrolled. All participants received treatment between December 2023 and November 2025, and completed at least one monitoring brain MRI scan prior to the fifth infusion. The eligibility criteria included the Mini-Mental State Examination (MMSE) score of 22 or higher and the Clinical Dementia Rating (CDR) global score of 0.5 or 1, confirmation of Aβ pathology by cerebrospinal fluid (CSF) biomarkers or amyloid PET, and absence of exclusionary MRI findings such as edema or effusion, five or more microhemorrhages, cortical superficial siderosis, or intracerebral hemorrhage >1 cm [7,8]. Baseline data included age, sex, MMSE, CDR and CDR Sum of Boxes (CDR-SB), *APOE* ε4 genotype, concomitant use of cholinesterase inhibitors, antiplatelet, anticoagulant, and antihypertensive drugs, and mean blood pressure (MBP) prior to the first infusion.

### 2.2 Ethical approval and consent

This study was performed in accordance with the tenets of the Declaration of Helsinki and was approved by the Institutional Review Board of the Tokyo Metropolitan Institute for Geriatrics and Gerontology (TMIG) (R23–117) and Tokyo Medical Biobank (R21–038). Written informed consents were obtained for CSF biomarker measurement, *APOE* ε4 genotyping, participation in Biobank, PET, Lecanemab treatment, and use of data from clinical practice.

### 2.3 MRI assessment

All MRI images were obtained on the same 3T MRI scanner (Ingenia Elition 3.0T, Koninklijke Philips NV, Amsterdam, the Netherlands). Diffusion-weighted images (DWI), susceptibility-weighted images (SWI), fluid-attenuated inversion recovery (FLAIR), T2-weighted images, and 3D T1-weighted images were obtained in accordance with the Japanese guidelines (**Supplement 1**) [12]. MRI contraindications, including microhemorrhages ≥5, macrohemorrhage, superficial siderosis, or vasogenic edema or effusion, were assessed by two expert neuroradiologists (F. S. and A. M. T.). Both completed the specific training course designated by the Japan Radiological Society, the Japanese Society for Magnetic Resonance in Medicine, and the Japanese Society for Neuroradiology [12]. These findings were also confirmed in a consensus conference attended by six neurologists, two expert neuroradiologists, one experienced nuclear medicine physician, and two psychiatrists. White matter hyperintensities on FLAIR images were graded in the periventricular (PV) and the deep white matter (DWM) using the Fazekas score [13].

All monitoring MRI scans were performed at our hospital using the same protocol. The schedule strictly followed the OUG (before the 5th, 7th, and 14th infusions, then every 6 months). This contrasts with US and French protocols, which mandate specific scans for *APOE* ε4 carriers (pre-26th or 27th) but do not require ongoing 6-month monitoring [14,15]. SWI was primarily used to detect ARIA-H, while FLAIR and DWI were utilized to identify and characterize ARIA-E. For patients who developed ARIA, imaging findings were shared and reviewed in a consensus conference, where the final interpretation was determined. The radiographic severity of ARIA was assessed in accordance with the appropriate use recommendations [14]. As this study was conducted for safety monitoring in a real-world clinical setting, neuroradiologists were not blinded to clinical information, including treatment status.

### 2.4 Amyloid PET scan

All amyloid PET scans conducted during this period used ^18^F-flutemetamol (Vizamyl®; Nihon Medi-Physics, Tokyo, Japan) or ^18^F-florbetapir (Amyvid®; PDR Pharma, Tokyo, Japan). PET images were obtained using an integrated PET/CT scanner, Discovery 710, and Discovery MI (GEHealthcare, Milwaukee, United States). Details of data acquisition and reconstruction methods were described elsewhere [16,17]. Amyloid positivity was determined by an expert visual reading (K. I) [18]. Centiloid (CL) scales were calculated as quantitative reference values: for ^18^F-flutemetamol, using a previous reported automated semi-quantitative analysis technique without anatomical images [17] provided in the VIZCalc software (Nihon Medi-Physics); for ^18^F-florbetapir, using AMYclz neuro software (PDR Pharma) utilizing both PET and CT scan/MRI data [19].

### 2.5 CSF biomarker testing

CSF was obtained via standard lumbar puncture. During the study period in Japan, Aβ_42_/Aβ_40_ ratio using the LUMIPULSE system (FUJIREBIO INC., Tokyo, Japan) and its assays were the only approved CSF assays to confirm eligibility for Lecanemab. The CSF concentrations of Aβ_42_, Aβ_40_, phosphorylated tau 181 (pTau181), and total tau (tTau) were measured using the LUMIPULSE system and its assays [20,21] at the central laboratory of SRL, Inc. (Tokyo, Japan). Predetermined cut-offs were 0.067 for Aβ_42_/Aβ_40_ [21], 56.5 pg/mL for pTau181, and 404 pg/mL for tTau [20].

### 2.6 Plasma biomarker testing

Stored plasma samples with sufficient residual volume in the Biobank were measured for neurofilament light chain (NfL) and glial fibrillary acidic protein (GFAP) using Simoa HD-X platform and Neurology 2-Plex B multiplex assay (Quanterix, Billerica, MA, USA) at Raybiotech, Inc. (Norcross, GA, USA) [18].

### 2.7 Safety

Safety assessments included amyloid-related imaging abnormalities (ARIA), infusion-related reactions (IRRs), and treatment discontinuation. IRRs were graded according to the Common Terminology Criteria for Adverse Event [22]. No premedication was administered for adverse event prevention.

### 2.8 Statistical methods

Categorical variables are expressed as percentages. Normally distributed continuous variables are expressed as mean ± standard deviation, whereas non-normally distributed continuous variables are expressed as median with interquartile range. Statistical significance of differences between patients with and without ARIA was assessed using *t*-tests and χ^2^ test. Additionally, relationships among baseline characteristics were evaluated using Pearson’s correlation coefficient, *t*-tests, and χ2 test. To achieve a normal distribution, GFAP and NfL were log10-transformed. For the exploratory analysis of ARIA risk factors, continuous variables were categorized according to prior studies or divided into subgroups of comparable size [11,23]. Unadjusted odds ratios (ORs) and 95% confidence intervals (CIs) were calculated. Statistical significance was set at *p* < 0.05. All statistical analyses were performed using IBM SPSS Statistics 29 (IBM Corp., Armonk, NY, USA).

### 2.9 Data availability statement

The data supporting the findings of this study are available from the corresponding author upon reasonable request.

## 3 Results

### 3.1 Characteristics of the patients

The 120 patients had a mean age of 74.2 ± 7.9 years, with 89 females (74%). The majority of patients (88%) had a baseline CDR of 0.5, and the mean CDR-SB score was 3.0 ± 1.4; 63% of the patients were diagnosed with MCI. Since *APOE* testing is not covered by the national insurance in Japan, data were available for 59 patients (49%). Nine patients (15%) were *APOE* ε4 homozygotes and 20 patients (34%) were ε4 heterozygotes. All patients underwent CSF testing (36 [30%]) or amyloid PET (84 [70%]) for confirmation of amyloid pathology. In 72 patients, plasma biomarker testing through the Biobank was performed. Baseline microbleeds were observed in 45 patients (38%). No patients exhibited cortical superficial siderosis. Fazekas score is not included in eligibility criteria in the Japanese OUGs; therefore, 38 patients (32%) had PV Fazekas score 2–3, and 27 patients (23%) had DWM Fazekas score 2–3. Fifteen patients (13%) were taking antiplatelet agents and four patients (3%) were receiving anticoagulant medications (Table 1). Regarding the relationships among baseline variables, apart from expected correlations within the same categories of biomarkers, age was significantly associated with amyloid PET CL (Pearson’s *r* = −0.25, *p* = 0.02), PV (*t*-test; *p* = 0.001), and DWM Fazekas scores (*t*-test; *p* = 0.04). No other significant associations were observed.

**Table 1:** Baseline characteristics of the participants with and without ARIA.

	Total (*n* = 120)	No ARIA (*n* = 96)	Any ARIA (*n* = 24)	*P*-value
Age (years)	74.2 ± 7.9	74.2 ± 8.3	74.2 ± 6.3	0.99^a^
Sex				
Female (%)	89 (74%)	72 (75%)	17 (71%)	0.68^b^
Male (%)	31 (26%)	24 (25%)	7(29%)	
MMSE	24.9 ± 2.1	25.1 ± 2.1	24.0 ± 1.9	0.02^a^*
CDR				
0.5	105 (88%)	81 (84%)	24 (100%)	0.04^b^*
1	15 (13%)	15 (16%)	0 (0%)	
CDR-SB	3.0 ± 1.4	3.0 ± 1.5	2.8 ± 1.1	0.60^a^
Clinical diagnosis				
MCI	76 (63%)	62 (65%)	14 (58%)	0.64^b^
Mild dementia	44 (37%)	34 (35%)	10 (42%)	
*APOE* ε4 status	*n*=59	*n*=43	*n*=16	
ε4 Homozygote	9 (15%)	6 (14%)	3 (19%)	0.46^b^
ε4 Heterozygote	20 (34%)	13 (30%)	7 (44%)	
ε4 Non-carrier	30 (51%)	24 (56%)	6 (38%)	
Amyloid PET Centiloid	*n*=82	*n*=70	*n*=12	
	60.8 ± 27.5	60.1 ± 27.8	65.0 ± 26.4	0.57^a^
CSF biomarkers	*n*=36	*n*=25	*n*=11	
Aβ_42/40_	0.048 ± 0.008	0.049 ± 0.007	0.046 ± 0.011	0.49^a^
p-tau181 (pg/mL)	94.7 ± 36.2	97.0 ± 35.2	89.4 ± 39.4	0.57^a^
t-tau (pg/mL)	638 ± 241	653 ± 211	604 ± 308	0.58^a^
Plasma biomarkers	*n*=72	*n*=55	*n*=17	
NfL (pg/mL)	22.5 (17.6–28.4)	22.0 (17.6–28.3)	24.3 (18.0–32.0)	0.23^a^
GFAP (pg/mL)	300 (237–424)	299 (233–409)	377 (254–507)	0.15^a^
Microbleeds				
0 (%)	75 (63%)	60 (63%)	15 (63%)	1.00^b^
1–4 (%)	45 (38%)	36 (37%)	9 (37%)	
PV Fazekas Score				
0–1 (%)	82 (68%)	68 (71%)	14 (58%)	0.24^b^
2–3 (%)	38 (32%)	28 (29%)	10 (42%)	
DWM Fazekas Score				
0–1 (%)	93 (78%)	78 (81%)	15 (63%)	0.05^b^*
2–3 (%)	27 (23%)	18 (19%)	9 (38%)	
Medication use				
Cholinesterase inhibitors (%)	43 (36%)	32 (33%)	11 (46%)	0.25^b^
Antiplatelet (%)	15 (13%)	12 (13%)	3 (13%)	1.00^b^
Anticoagulant (%)	4 (3%)	3 (3%)	1 (4%)	0.80^b^
Antihypertensive (%)	54 (45%)	45 (47%)	9 (38%)	0.41^b^
Mean arterial blood pressure (mmHg)	93.9 ± 12.7	93.0 ± 13.0	97.3 ± 11.0	0.14^a^

Values are expressed as means ± standard deviations or medians (interquartile ranges).

*P*-values represent the result of: a, Student’s *t*-test; b, χ^2^ test.

*P-value <0.05.

Abbreviations: ARIA, amyloid-related imaging abnormalities; MMSE, Mini-Mental State Examination; CDR, Clinical Dementia Rating; CDR-SB, Clinical Dementia Rating Sum of Boxes; MCI, mild cognitive impairment; NfL, neurofilament light chain; GFAP, glial fibrillary acidic protein; PV, periventricular; DWM, deep white matter.

### 3.2 Flowchart of treatment status

**Fig. 1:**
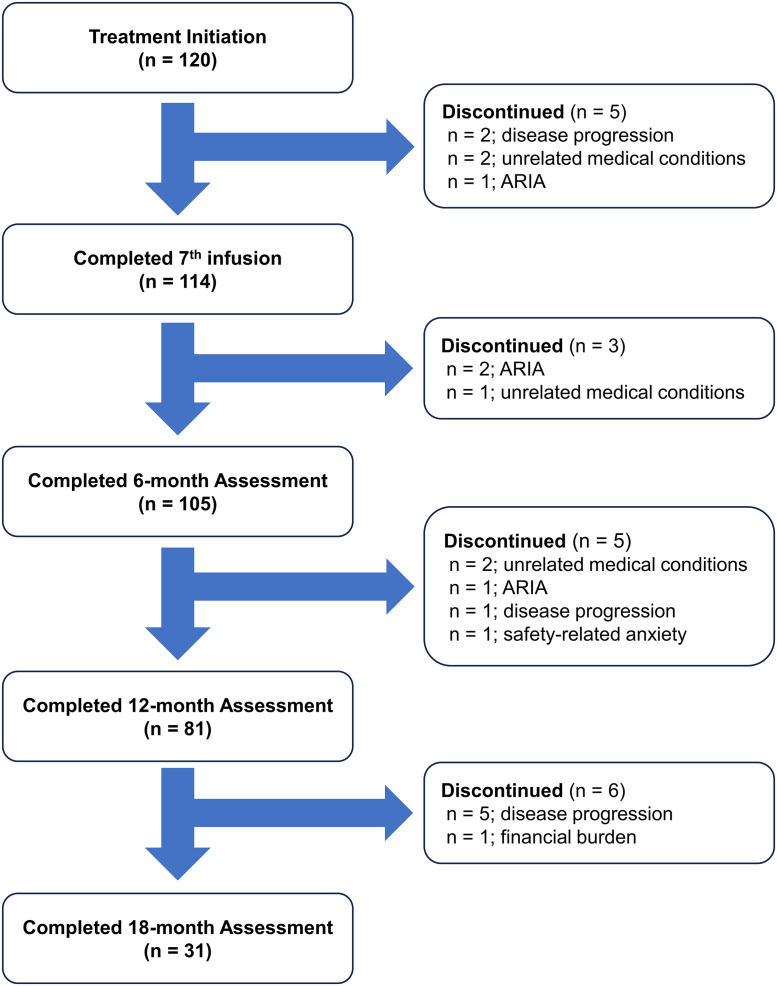
Flowchart of treatment status in patients.

As of November 2025, 81 of 120 patients had completed the 12-month assessment. During the study period, 19 patients (16%) discontinued treatment: eight due to disease progression, five due to unrelated medical conditions, four due to ARIA, one due to safety-related anxiety, and one due to financial burden (Fig. 1). ARIA was a more common cause of early discontinuation, whereas disease progression predominated in later discontinuation.

### 3.3 Amyloid-Related imaging abnormalities

**Fig. 2:**
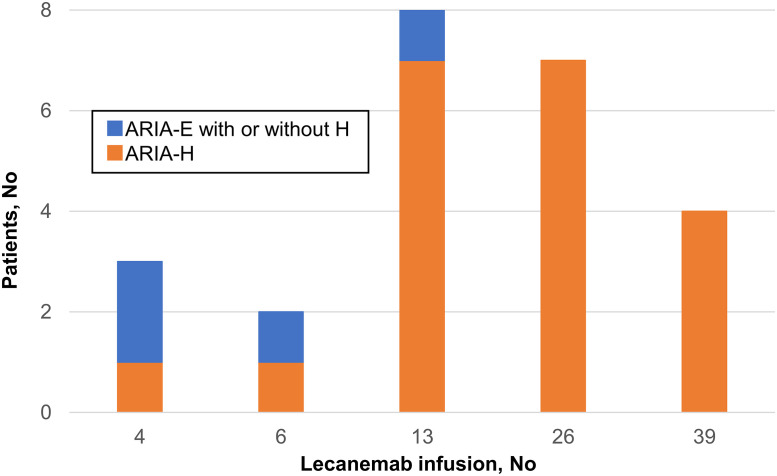
Timing and severity of amyloid-related imaging abnormalities. (A) Number of infusions to first amyloid-related imaging abnormalities in Lecanemab treated patients. The blue bars represent the number of ARIA-E with or without ARIA-H, and the orange bars represent the number of isolated ARIA-H. The numbers of patients in the table represent those who underwent MRI, excluding patients who had developed ARIA up to that time. (B) The radiographic severity of ARIA was assessed in accordance with the appropriate use guidelines. Orange dots represent continued cases and blue dots represent discontinued cases.

Abbreviations: ARIA-E, amyloid-related imaging abnormalities cerebral edema or effusion; ARIA-H, amyloid-related imaging abnormalities cerebral hemorrhage or hemosiderin deposition.

ARIA occurred in 24 of 120 patients (20%); ARIA-E with ARIA-H occurred in four patients (3%), ARIA-E without ARIA-H occurred in one patient (1%), and isolated ARIA-H occurred in 19 patients (16%). All were asymptomatic. Among the five patients with ARIA-E, events occurred within 6 months of treatment initiation. In contrast, the occurrence of isolated ARIA-H gradually increased over time, with 1% (1/120) within the first 2 months, 1% (1/114) between 2 and 3 months, 6% (6/95) between 3 and 6 months, 9% (7/74) between 6 and 12 months, and 16% (4/25) between 12 and 18 months after treatment initiation (Fig. 2A). Radiographic assessment showed that most ARIA cases were mild (21 of 24 [88%]), predominantly occurring as isolated mild ARIA-H or ARIA-E (19 of 24 [79%]) (Fig. 2B).

Four patients discontinued treatment due to ARIA (Fig. 2B, Table 2). In Patient 1, mild ARIA-E and moderate ARIA-H were observed before the fifth infusion, leading to treatment interruption. A follow-up MRI performed two months later showed an increase in the number of microhemorrhages to >10 microhemorrhages, prompting permanent discontinuation. One month thereafter, the findings had stabilized. In Patient 2, mild ARIA-E was observed before the seventh infusion. While the treatment was continued, the severity had progressed to moderate before the ninth infusion, leading to treatment interruption. One month later, MRI showed two new microhemorrhages. On MRI performed one additional month later, the findings had stabilized, and treatment was resumed; however, moderate ARIA-E and mild ARIA-H recurred at the same site, resulting in treatment discontinuation after discussion with the patient. Stabilization was confirmed two months later. In Patient 3, emergency surgery for aortic dissection was performed before the tenth infusion. After surgery, one antiplatelet agent and one anticoagulant were initiated. Prior to Lecanemab treatment resumption, MRI revealed more than 10 microhemorrhages, leading to treatment discontinuation. Stabilization was confirmed three months later. In Patient 4, one new microhemorrhage in the right frontal lobe was detected before the seventh infusion. MRIs before the tenth and twelfth infusions showed one additional microhemorrhage and cortical hemosiderin deposition, remaining classified as mild ARIA-H. Before the fourteenth infusion, two new microhemorrhages and mild ARIA-E were observed, and the ARIA-H was ultimately classified as moderate, leading to treatment discontinuation. Although MRI two months later showed stabilization, treatment was not resumed after careful discussion with the patient.

**Table 2:** Discontinued patients due to amyloid-related imaging abnormalities.

	Sex/age	Baseline MMSE/CDR	Baseline Microbleeds number	Baseline Fazekas PV/DWM	Anticoagulant/ Antiplatelet use	Number of infusions to first ARIA	ARIA-E	ARIA-H
Patient 1	M/50s	23/0.5	1	0/0	no	4th	mild	moderate
Patient 2	F/70s	26/0.5	1	1/1	no	6th	moderate	mild
Patient 3	M/70s	23/0.5	0	1/1	antiplatelet	9th	no	severe
Patient 4	F/70s	24/0.5	1	1/2	no	6th	mild	moderate

ARIA severity represents the maximal radiographic severity.

Abbreviations: M, Male; F, Female; MMSE, Mini-Mental State Examination; CDR, Clinical Dementia Rating; PV, periventricular; DWM, deep white matter; ARIA-E, amyloid-related imaging abnormalities cerebral edema or effusion; ARIA-H, amyloid-related imaging abnormalities cerebral hemorrhage or hemosiderin deposition.

The patient characteristics with and without ARIA are presented in Table 1. Patients with ARIA had significantly lower baseline MMSE scores than those without ARIA (24.0 ± 1.9 vs. 25.1 ± 2.1, *p* = 0.02). In contrast, the proportion of patients with CDR of 0.5 was significantly higher in the ARIA group (100% vs. 84%, *p* = 0.04). The prevalence of DWM Fazekas grade 2–3 was also significantly higher among patients with ARIA (38% vs. 19%, *p* = 0.05). The frequency of PV Fazekas grade 2–3 (42% vs. 29%, *p* = 0.24), mean arterial blood pressure (97.3 ± 11.0 vs. 93.0 ± 13.0, *p* = 0.14), and plasma biomarker levels including NfL and GFAP (*p* = 0.23 and 0.15, respectively) were all numerically higher in the ARIA group; however, these differences did not reach statistical significance. In this cohort, *APOE* genotype data were available for 49% of the patients. Given the substantial missing data and limited statistical power, the association between *APOE* ε4 status and ARIA risk was inconclusive in this study (*p* = 0.46). There were no significant differences between patients with and without ARIA in baseline microhemorrhages, amyloid PET CL, or the use of antiplatelets, anticoagulants, or antihypertensive medications.

When analyses were restricted to ARIA-E with or without ARIA-H, there was a non-significant trend toward an association with the number of *APOE* ε4 alleles (χ^2^ test; *p* = 0.10). Among the five patients who developed ARIA-E with or without ARIA-H, *APOE* genotype data were available for four. Of these, one was an *APOE* ε4 homozygote and three were heterozygotes. When analyses were further restricted to isolated ARIA-H, the proportion of patients receiving antihypertensive medications was lower in the ARIA-H group (26%) than in the no ARIA group (47%) (χ^2^ test; *p* = 0.10), suggesting a potential trend toward a lower incidence of isolated ARIA-H among patients taking antihypertensive medications.

**Fig. 3:**
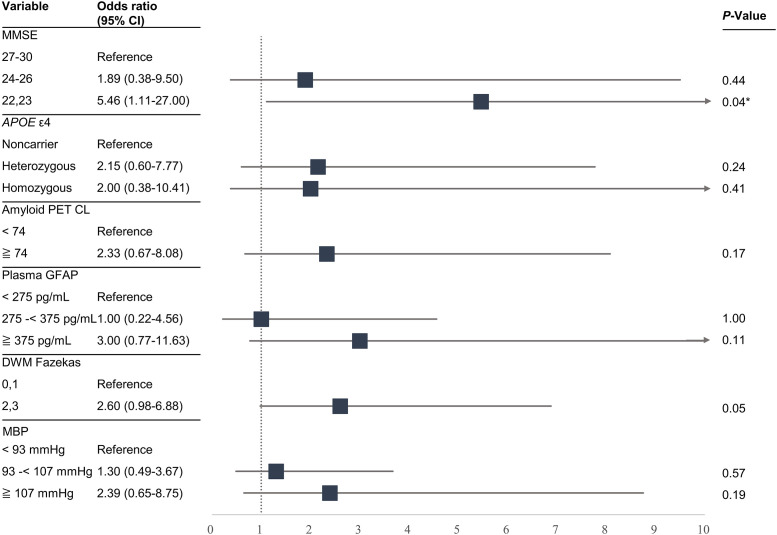
Unadjusted odds ratios of baseline risk factors associated with amyloid-related imaging abnormalities. Forest plot showing the association with ARIA. Continuous variables were categorized according to prior studies or divided into subgroups of comparable size. Unadjusted odds ratios and 95% confidence intervals were calculated. *P*-values were determined using the χ² test. *P-value <0.05. Abbreviations: MMSE, mini-mental state examination; CL, Centiloid; GFAP, glial fibrillary acidic protein; DWM, deep white matter; MBP, mean arterial blood pressure.

Based on the group comparisons described above and prior studies [11,23], six baseline factors were selected for further exploratory analysis regarding ARIA risk. In this analysis, continuous variables were categorized into subgroups, and unadjusted odds ratios were calculated. Lower MMSE scores were significantly associated with an increased risk of ARIA compared with higher scores (22,23 vs 27–30: OR, 5.46; 95% CI, 1.11–27.00; *p* = 0.04). Higher GFAP levels (≥ 375 pg/mL vs < 275 pg/mL: OR, 3.00; 95% CI, 0.77–11.63; *p* = 0.11) and higher DWM Fazekas scores (2–3 vs 0–1: OR, 2.60; 95% CI, 0.98–6.88; *p* = 0.05) yielded numerically higher odds; however, these estimates did not reach statistical significance. Greater amyloid PET CL (≥ 74 CL vs <74 CL: OR, 2.33; 95% CI, 0.67–8.08; *p* = 0.17) and higher mean arterial blood pressure (≥107 mmHg vs <93 mmHg: OR, 2.39; 95% CI, 0.65–8.75; *p* = 0.19) showed increases in the same direction but did not reach statistical significance. Overall, the CIs were relatively wide, reflecting the limited sample size (Fig. 3).

### 3.4 Infusion-Related reactions

IRRs occurred in 34 of 120 patients (28%), all of which were mild. The most frequently reported symptom was fever (25 of 34, 74%), followed by fatigue, headache, and nausea. Symptoms emerged during the first infusion in all patients, and in some cases persisted through the eighth infusion (**Supplement 2**). Patients who developed IRRs at the first infusion received oral acetaminophen prior to subsequent infusions. These reactions did not lead to discontinuation of treatment in any patient. Adverse events were assessed at every visit. As a serious adverse event, one patient was hospitalized overnight on the first infusion day related to an IRR, and the symptoms resolved within 24 h.

## 4 Discussion

The primary aim of this study was to evaluate the safety of treating Japanese patients with Lecanemab at the single center. Overall, our findings suggest that Lecanemab therapy can be administered with a manageable safety profile in a real-world Japanese clinical setting. No patients developed symptomatic ARIA, and all cases with ARIA stabilized without major complications. This may be attributable to careful patient selection in accordance with the OUGs and vigilant monitoring of MRI scans for ARIA. Furthermore, similar to recent reports from China where no symptomatic ARIA was observed [23], population-level factors in East Asian cohorts (e.g., genetic background and clinical characteristics) may be associated with differences in the symptomatic presentation of ARIA.

The incidence of IRRs was 28%, which is consistent with the 26% observed in the Clarity-AD trial [4] and the 37% reported in a real-world study from Washington University in St. Louis [11]. Notably, the Clarity-AD trial’s subgroup analysis indicated a lower incidence of 12% in the Asian population [5]. However, our study did not observe this trend, as the IRR rate in our cohort was comparable to that of the overall Clarity-AD population. All IRRs were mild and were managed with oral acetaminophen alone. These reactions did not result in treatment discontinuation and were considered manageable.

The incidence of ARIA-E was 4%, whereas previous studies of Lecanemab have reported a higher incidence of ARIA-E, including 13.6% in the Clarity-AD trial [4,6,11]. One possible explanation for this is the lower prevalence of *APOE* ε4 carriers (homozygous: 15%, heterozygous: 34%), as *APOE* ε4 status has been identified as a significant risk factor for ARIA-E. Indeed, in our cohort, all ARIA-E positive patients with available *APOE* data were homozygous or heterozygous carriers. Also, there was a suggestive trend between ARIA-E and the number of *APOE* ε4 alleles. However, the Clarity-AD trial reported a similarly low incidence of ARIA-E in the Asian subgroup (6.2%), despite comparable *APOE* ε4 carrier frequencies between the Asian (72.6%) and overall (69.0%) populations [5]. These findings raise the possibility that Asian populations may have a lower risk of ARIA-E irrespective of *APOE* ε4 status. Another potential mechanism for the low ARIA-E rate may involve differences in amyloid PET burden. In the TRAILBLAZER-ALZ and ALZ 2 trials of donanemab, a significant association between amyloid PET burden and ARIA-E risk has been observed [23]. In our cohort, amyloid PET burden was relatively low (62.3 ± 24.3), which may partly explain the lower ARIA-E risk, although a relationship between amyloid PET burden and ARIA-E has not been reported for Lecanemab [6].

ARIA-H has been reported in placebo groups, reflecting its natural occurrence, with an incidence of 7.8% in the Clarity-AD trial and 7.0% in the Japanese subgroup of the TRAILBLAZER-ALZ 2 trial [4,25]. In our cohort, isolated ARIA-H gradually increased over time. While this accumulation may partly reflect the natural history of microhemorrhages, the overall incidence of 19 patients (16%) was notably higher than that observed in the placebo groups of these trials. In the Asian subgroup of the Clarity-AD trial, the incidence was lower and comparable to placebo [5], suggesting that the elevated rate in our study is unexpected. One possible explanation is that our cohort had a higher prevalence of baseline microhemorrhages and included patients with severe white matter lesions who were excluded from clinical trials, and the inclusion of such patients with a higher vascular risk profile may have influenced the incidence of ARIA-H. However, contrary to clinical trial findings, baseline microhemorrhages were not associated with ARIA in our cohort. This discrepancy may stem from the restricted analytical range (0 to 4 microhemorrhages) resulting from the strict exclusion of patients with ≥5 microhemorrhages or limited statistical power. Another explanation is the higher sensitivity for detecting microhemorrhages at our institution, attributable to the use of SWI in our study, whereas T2* gradient recalled echo was used in the Clarity-AD trial [26]. In addition, a study comparing AI-assisted readings with radiologist assessments has shown that up to 40% of mild ARIA-H cases can be missed by radiologists, indicating that differences in detection capabilities among radiologists may also influence the reported incidence of ARIA-H [27,28].

Compared with patients without ARIA, those with ARIA had lower baseline MMSE scores. This finding is consistent with a previous real-world study [11] and may reflect an increased burden of cerebral amyloid angiopathy associated with more advanced AD [10]. In contrast, the proportion of patients with a CDR of 0.5 was higher among those with ARIA. A discrepancy between MMSE and CDR scores was observed, which may be attributable to the limited sample size and potential selection bias, or to the influence of other pathologies on CDR assessments. Although not statistically significant, the numerically higher frequency of ARIA in patients with a baseline CL ≥ 74 warrants further investigation. Notably, in this cohort, no patients with CL ≤ 25 (*n* = 8) developed ARIA.

A numerically higher frequency of ARIA was observed in patients with higher baseline plasma GFAP levels, suggesting a potential role of astrocytes in ARIA occurrence. Astrocytes play a critical role in maintaining blood brain barrier (BBB) integrity [29,30], and their activation is reflected by elevated GFAP expression [31]. Reactive astrogliosis, which may occur early in relation to classical AD pathologies such as Aβ deposition and tau aggregation [32], has been linked to BBB disruption through neuroinflammatory processes [33]. Increased BBB leakage has been implicated in the formation of microhemorrhages [[34], [35], [36], [37]], suggesting a potential pathway by which elevated GFAP levels might be linked to ARIA occurrence in AD. However, if astrocyte-driven BBB disruption was the primary mechanism, baseline GFAP levels would be expected to correlate with pre-treatment number of microhemorrhages, which was not observed, suggesting that other mechanisms may also contribute. Another possibility is that baseline GFAP levels correlate with Aβ accumulation [38,39], raising the question of whether the relationship between Centiloid and ARIA might be mediated by GFAP. Furthermore, given the known correlation between GFAP levels and MMSE scores in AD [40], GFAP might also mediate the relationship between MMSE scores and the occurrence of ARIA.

The potential link between higher DWM Fazekas scores and ARIA suggested in our exploratory analysis is consistent with previous real-world reports demonstrating a relationship between Fazekas score and ARIA [24,41]. A prior study has demonstrated a strong association between white matter hyperintensity (WMH) and microhemorrhages, and has suggested that WMH may represent an early manifestation of vascular amyloidosis preceding the development of microhemorrhages [42]. However, in accordance with the OUGs, patients presenting with severe hemorrhagic or edematous abnormalities defined as contraindications are excluded from Lecanemab treatment. Since this effectively rules out cases with severe cerebral amyloid angiopathy (CAA), the WMH observed in eligible patients likely reflects a composite of early-stage amyloid pathology and non-amyloid vascular factors, such as hypertensive arteriosclerosis. This superimposition of multiple risk factors may contribute to an underlying vascular vulnerability that may be associated with a higher susceptibility to ARIA. Elevated mean blood pressure tended to be associated with an increased risk of ARIA, whereas the use of antihypertensive medications tended to be associated with a reduced risk of ARIA-H, suggesting a potential contribution of vascular risk factors to ARIA susceptibility. To ensure stable control, we monitor blood pressure at every clinic visit and, when available, refer to home blood pressure records. When hypertension is identified or existing control is inadequate, we either initiate antihypertensive therapy or adjust current medications before starting Lecanemab. These findings underscore the importance of blood pressure management before treatment initiation. Although anti-amyloid antibody treatment is generally not recommended for patients receiving anticoagulants [14], four patients who were taking anticoagulants were treated with Lecanemab in this cohort. At present, no significant association has been observed between anticoagulant use and the occurrence of ARIA. However, as a notable exception, one patient developed more than 10 microhemorrhages. Because this patient had initiated an anticoagulant following surgery for an aortic dissection, and major cardiac surgery itself significantly increases the risk of new microhemorrhages [43], it is difficult to attribute the ARIA solely to the medication. Therefore, the surgical intervention and subsequent anticoagulant use likely contributed synergistically to the severity of ARIA in this case.

Nineteen patients discontinued treatment, 53% of whom discontinued due to disease progression. Lecanemab treatment was frequently discontinued in patients who progress to moderate dementia, reflecting patient and family preferences. In addition, some patients discontinued treatment at an earlier stage, for example due to the emergence of behavioral and psychological symptoms of dementia (BPSD). Four patients discontinued treatment due to ARIA. Excluding one patient who underwent surgery for aortic dissection during the treatment period, the initial ARIA events in the remaining three patients occurred before the seventh infusion. Among patients who developed ARIA before the seventh infusion but did not discontinue treatment, follow-up MRI after ARIA onset demonstrated rapid stabilization. In contrast, in patients who eventually discontinued treatment, worsening was observed on follow-up MRI, even when treatment was not immediately discontinued. Patients 2 and 4 exhibited opposite temporal sequence of ARIA subtypes: In Patient 2, ARIA-E emerged first, followed by ARIA-H in the same region; in Patient 4, ARIA-H appeared first, followed by ARIA-E in the same region. These observations suggest that early emergence of either subtype may precede the other or herald overall worsening over a short interval. Regardless of ARIA subtype, the early occurrence of ARIA was associated with a higher risk of treatment discontinuation, and particular caution may be warranted when ARIA does not stabilize promptly.

The primary limitation of this study is the lack of a control group, which complicates the differentiation between disease-related progression and treatment-related effects. In addition, plasma biomarkers were not obtained for all patients, and *APOE* ε4 status, which is not covered by insurance, was available in only approximately half of the cohort; therefore, comprehensive biomarker data were not available. Notably, the EMA recommended marketing authorisation for Lecanemab only in patients with one or no copy of *APOE* ε4 (thus excluding ε4 homozygotes). In contrast, because *APOE* genotyping has not been reimbursed under Japan’s national health insurance system and is not routinely implemented in clinical practice, the Japanese guidelines include no *APOE*-related restrictions on Lecanemab use. Given the small number of homozygotes in our study, the risks of ARIA and severe adverse events in this high-risk population may be underestimated. Together with the relatively small sample size, this limitation indicates that further accumulation of cases will be necessary. Finally, several risk factors identified in this study, such as baseline MMSE, GFAP levels, Fazekas score and amyloid burden, are known to be biologically interrelated. Due to the limited number of ARIA events, multivariate analysis to adjust for confounding was not feasible. Therefore, we could not determine the independent contribution of each factor to ARIA risk.

In this Japanese AD cohort, Lecanemab demonstrated a manageable safety profile in a real-world setting, supporting its feasibility in routine clinical practice. While the incidence of ARIA-E was lower than in clinical trials, potentially reflecting a lower risk profile in Asian populations, isolated ARIA-H occurred more frequently, likely reflecting a higher baseline vascular burden in this real-world population. In exploratory analyses, potential trends toward a higher frequency of ARIA were observed in patients with lower MMSE scores, higher plasma GFAP levels, and higher Fazekas scores, underscoring the importance of individualized risk assessment prior to therapy.

## Funding agencies

This study was supported by JSPS KAKENHI Grant Number JP24K10653 (RI), Translational Research Grant from the TMIG to MK, AMED Grant Number JP24he2202020 (AI), the Integrated Research Initiative for Living Well with Dementia (IRIDE) and the Program for Supporting Antibody Therapies for Dementia (English translation of 東京都認知症抗体医薬対応支援事業) from the Tokyo Metropolitan Government.

## Declaration of generative AI and AI-assisted technologies in the manuscript preparation process

The authors used Gemini 3 Pro (Google LLC) for English language editing of the manuscript. After using this tool/service, the authors reviewed and edited the content as needed and take full responsibility for the content of the published article.

## CRediT authorship contribution statement

**Ryosuke Shimasaki:** Writing – review & editing, Writing – original draft, Visualization, Software, Methodology, Investigation, Formal analysis, Data curation, Conceptualization. **Masanori Kurihara:** Writing – review & editing, Resources, Methodology, Investigation, Funding acquisition, Data curation, Conceptualization. **Taro Bannai:** Writing – review & editing, Investigation, Data curation. **Keiko Hatano:** Writing – review & editing, Investigation, Data curation. **Fumio Suzuki:** Writing – review & editing, Investigation, Data curation. **Aya Midori Tokumaru:** Writing – review & editing, Investigation, Data curation. **Kenji Ishii:** Writing – review & editing, Investigation, Data curation. **Ryoko Ihara:** Writing – review & editing, Writing – original draft, Validation, Supervision, Resources, Project administration, Methodology, Investigation, Funding acquisition, Data curation, Conceptualization. **Atsushi Iwata:** Writing – review & editing, Supervision, Resources, Funding acquisition.

## Declaration of competing interest

The authors declare the following financial interests/personal relationships which may be considered as potential competing interests: MK received honoraria for lectures from Eisai, FUJIREBIO and Nihon Medi-Physics; and patent assignment fee from FUJIREBIO. TB received honoraria for lectures from Eisai. KI received advisory fees, honoraria for lectures and research grants from Eli Lilly, Nihon Medi-Physics and PDR Pharma. RI received advisory fees from Eisai, Eli Lilly and MSD; consultant fee from Chugai; and honoraria for lectures from Eisai, Eli Lilly, Nihon Medi-Physics, PDR Pharma, FUJIREBIO, Sysmex and IQVIA. AI received research grants from Eisai, FUJIREBIO, Janssen pharma, Sysmex, Kobayashi Pharma, Eli Lilly, Fujifilm, SONY, Biogen and Chugai/Roche; advisory fees from Eisai, FUJIREBIO, Eli Lilly, Roche, GSK, Otsuka, Soundwave Innovation; honoraria for lectures from Eisai, Eli Lilly, Biogen, Chugai/Roche, HU frontier, FUJIREBIO, Kowa, Sysmex, Ono, Otsuka, Alnylam, Daiichi Sankyo, Tokio Marine & Nichido Fire Insurance, PDR pharma, IQVIA, Sumitomo Pharma, MSD, Janssen pharma, and Kyowa Kirin; patent assignment fee from FUJIREBIO; and is involved in postmarketing surveillance of Lecanemab in Japan. This manuscript has been prepared in a neutral and objective manner, and all disclosed financial relationships are not relevant to the content of this work.

## Appendix Supplementary materials

Image, application 1
