# Supplementary material for: Amyloid-related imaging abnormalities in Japanese patients with Alzheimer’s disease treated with Lecanemab: A real-world study
Source: J Prev Alzheimers Dis. 2026 Apr 4;13(6):100562. doi: 10.1016/j.tjpad.2026.100562 (PMC13090307; doi:10.1016/j.tjpad.2026.100562)
Supplement: Supplementary file 1 [file mmc1.docx]

**Supplement 1.** MRI Sequence

|  | **DWI** | **3D T1** | **T2** | **FLAIR(Ax)** | **SWI** | **FLAIR(Cor)** |
| --- | --- | --- | --- | --- | --- | --- |
| Repetition Time (ms) | 4000 | 6.7 | 4000 | 11000 | 31 | 10000 |
| Echo Time (ms) | 72 | 3.9 | 90 | 120 | 6.2 | 75 |
| Inversion Time (ms) |  |  |  | 2700 |  | 3300 |
| Bandwidth (Hz/Px) | 27.8 | 434 | 411 | 579 | 289 | 391 |
| Number of Signals Averaged | 1 | 1 | 2 | 1 | 1 | 2 |
| Matrix | 132x129 | 256x200 | 368x280 | 332x225 | 340x308 | 296x230 |
| Field of View (mm) | 220 | 256 | 220 | 220 | 220 | 200 |
| Slice Thickness (mm) | 5 | 1 | 5 | 5 | 1 | 3 |
| Scan Time (min) | 1:08 | 5:14 | 2:33 | 2:45 | 2:27 | 5:40 |

**Supplement 2.** Number of Patients with Infusion-Related Reactions by Infusion Number


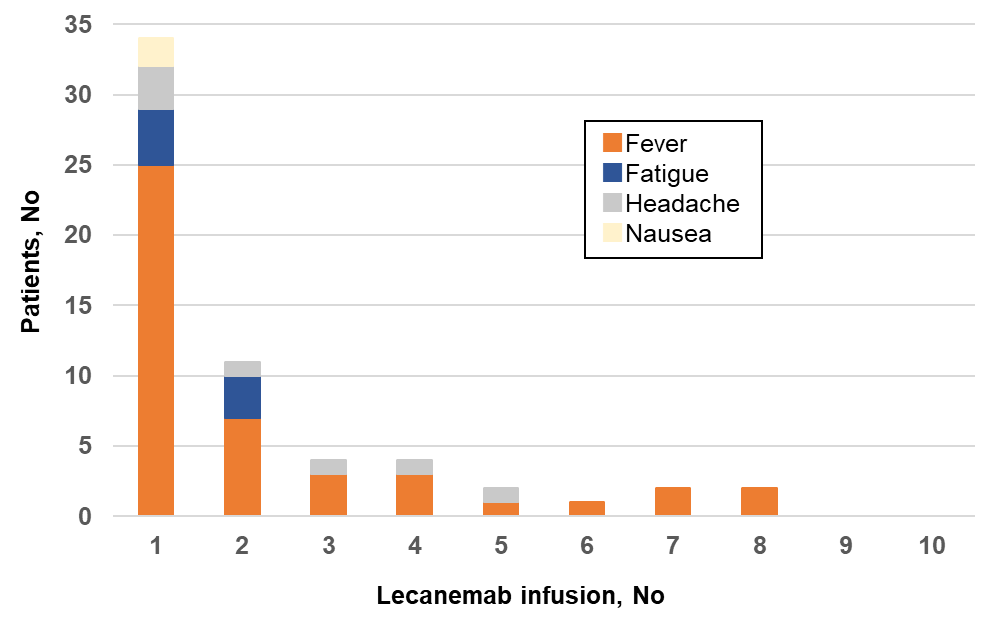


Patients were counted repeatedly if infusion-related reactions occurred at multiple infusions.
